# Shared antibiotic resistance and virulence genes in *Staphylococcus aureus* from diverse animal hosts

**DOI:** 10.1038/s41598-022-08230-z

**Published:** 2022-03-15

**Authors:** Spencer A. Bruce, Joshua T. Smith, Jennifer L. Mydosh, John Ball, David B. Needle, Robert Gibson, Cheryl P. Andam

**Affiliations:** 1 Department of Biological Sciences, University at Albany, State University of New York, Albany, NY 12222 USA; 2 Department of Molecular, Cellular and Biomedical Sciences, University of New Hampshire, Durham, NH 03824 USA; 3 Infectious Disease and Microbiome Program, Broad Institute of MIT and Harvard, Cambridge, MA 02142 USA; 4 New Hampshire Veterinary Diagnostic Laboratory, Durham, NH 03824 USA

**Keywords:** Antimicrobial resistance, Genetic variation, Microbiology, Population genetics

## Abstract

The emergence of methicillin-resistant *Staphylococcus aureus* (MRSA) poses an important threat in human and animal health. In this study, we ask whether resistance and virulence genes in *S. aureus* are homogeneously distributed or constrained by different animal hosts. We carried out whole genome sequencing of 114 *S. aureus* isolates from ten species of animals sampled from four New England states (USA) in 2017–2019. The majority of the isolates came from cats, cows and dogs. The maximum likelihood phylogenetic tree based on the alignment of 89,143 single nucleotide polymorphisms of 1173 core genes reveal 31 sequence types (STs). The most common STs were ST5, ST8, ST30, ST133 and ST2187. Every genome carried at least eight acquired resistance genes. Genes related to resistance found in all genomes included *norA* (fluoroquinolone)*, arlRS* (fluoroquinolone)*, lmrS* (multidrug)*, tet(38)* (tetracycline) *and mepAR* (multidrug and tigecycline resistance). The most common superantigen genes were *tsst-1*, *sea* and *sec*. Acquired antibiotic resistance (*n* = 10) and superantigen (*n* = 9) genes of *S. aureus* were widely shared between *S. aureus* lineages and between strains from different animal hosts. These analyses provide insights for considering bacterial gene sharing when developing strategies to combat the emergence of high-risk clones in animals.

## Introduction

*Staphylococcus aureus* is notable for its ability to colonize a wide range of vertebrate hosts, with each host representing a distinct ecological niche. In humans, it is responsible for severe nosocomial and community-associated infections^1–3^. *S. aureus* infections in animals are most commonly reported as a cause of mastitis in dairy-producing animals (including cattle, sheep and goats), osteomyelitis in poultry, and skin abscesses, mastitis and septicemia in farmed rabbits^4^. *S. aureus* has been reported in companion animals (e.g., dogs, cats)^5,6^ and wildlife^7,8^. Animal-associated *S. aureus* is also known to carry diverse antibiotic resistance genes and can therefore act as a reservoir for transmission of resistant strains to humans^9^. The emergence and spread of multidrug-resistant and methicillin-resistant *S. aureus* (MRSA) are therefore not limited to hospital and community settings, but also pose an important threat in veterinary medicine, agricultural systems and food production. Therefore, understanding the population and genome dynamics of MRSA across multiple eukaryotic hosts is key to advancing a One Health approach to attain optimal health for people, domestic animals, wildlife, plants, and the environment^10^

The ability to switch and adapt to new hosts are a dynamic and a constant feature of the evolution of *S. aureus*. Some host transitions are particularly notable. Two lineages in the clonal complex (CC) 97 that cause epidemic community-associated MRSA in humans was inferred to have evolved from independent bovine-to-human host jumps more than 40 years ago^11^. Methicillin resistance in this clone was acquired after the host-jump, likely as a result of strong selective pressures imposed by the widespread use of antibiotics for treating human infections^11^. Host-jumping events can also happen from humans to animals, as in the case of sequence type 5 (ST5) in broiler chickens that jumped from humans in the early 1970s^12^. Another lineage, CC398, has jumped from humans to livestock and back to humans, with resistance to methicillin and tetracycline acquired after the introduction to livestock from humans^13^. The majority of healthcare-associated infections of livestock-associated MRSA CC398 in Denmark from 2014 to 2016 were shown to be the result of repeated spillover introductions from nearby pig farms into healthcare facilities^14^. Human-to-monkey jumps of *S. aureus* ST6 and ST15 that occurred 2700 years ago was facilitated by the loss of phage-carrying genes that played a role in human colonization^15^. Over the past 5000–6000 years, numerous instances of cross-species transmission events have occurred in *S. aureus*^16^. These host-species transitions have been associated with horizontal gene transfer (HGT) of genetic elements conferring traits required for survival in the new host^16^. Some of these cross-species jumps have resulted in the emergence of successful outbreak-causing and widely circulating *S. aureus* lineages in humans and animals^16^.

The generalist nature of a pathogen can provide many opportunities for mobilizable DNA to be disseminated more widely among strains that colonize different hosts. However, it remains unclear whether for multi-host bacteria such as *S. aureus*, horizontally transferred genes follow the distribution of the bacterial cell that harbor them or create their own distribution. In this study, we sought to elucidate the population genomic structure of clinical animal-associated *S. aureus*. We ask whether the gene pool of resistance and virulence in *S. aureus* is homogeneously distributed or constrained by different animal hosts. Using whole genome sequencing of 114 *S. aureus* isolates from ten animal species, we demonstrate that a remarkable array of acquired resistance genes and superantigen genes of *S. aureus* are widely shared between multiple *S. aureus* lineages and between strains from different animal hosts. This study provides important insights for considering bacterial gene sharing between different animal hosts in developing strategies to combat the emergence of high-risk clones in animals.

## Results

### Phylogenetic diversity of animal-associated *S. aureus* in New England

**Figure 1:**
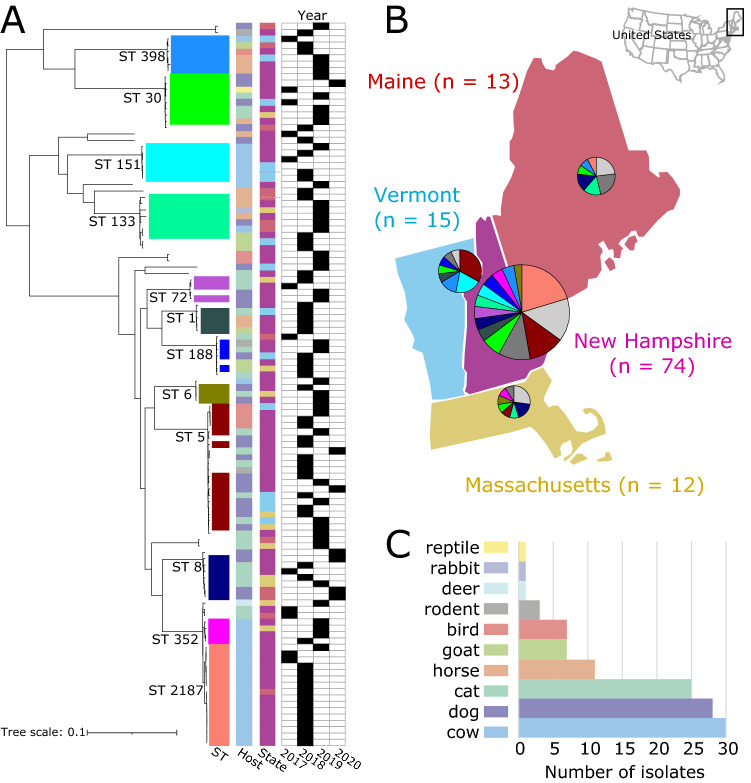
Phylogenetic diversity and sources of animal-associated *S. aureus* in New England. (**A**) Midpoint-rooted maximum likelihood phylogenetic tree. Tree scale represent the number of substitutions per site. For visual clarity, only those STs with at least three isolates are labeled. (**B**) Geographical distribution of STs. Pie charts show the proportion of major STs shown in panel A. Light gray includes STs consisting of a single isolate and novel STs, while dark gray represents STs consisting of two isolates. The number in parentheses indicates the number of isolates from each state. Maps were created using QGIS v3.22 (https://www.qgis.org/en/site/) (**C**) Number of isolates from different animal hosts.

We obtained a total of 114 high quality draft genomes of *S. aureus* isolates obtained through routine diagnostic tests of clinical specimens from diseased animals. These were submitted to the New Hampshire Veterinary Diagnostic Laboratory (NHVDL) from October 2017 to October 2019 (Fig. 1A and Supplementary Table S1). We obtained the isolates from four states in the United States: New Hampshire (*n* = 74), Maine (*n* = 13), Massachusetts (*n* = 12) and Vermont (*n* = 15) (Fig. 1B). The majority of isolates came from cows (*n* = 30), dogs (*n* = 28) and cats (*n* = 25) (Fig. 1C). Other domestic animals from which we obtained isolates included horses, goats and rabbits, while wild animals included birds, rodents, deer, rabbits and a tortoise.

De novo assembly of the 114 genomes generated sequences of sizes ranging from 2.68 to 2.90 Mb (mean = 2.77 Mb). The number of predicted genes ranged from 2429 to 2739 per genome (mean = 2570) (Supplementary Table S1). The pan-genome of the New England *S. aureus* population consisted of 7500 orthologous gene families. The genes in the pan-genome were categorized into core genes (present in 99% of genomes), soft-core genes (present in 95% to < 99% of genomes), shell genes (present in 15% to < 95% of genomes), and cloud genes (present in < 15% of genomes). We identified 1773 core genes (present in 113–114 genomes), 116 soft core genes (present in 109–112 genomes), 1158 shell genes (present in 18–108 genomes) and 4453 cloud genes (present in 1–17 genomes) (Supplementary Table S2). The combined core and soft-core genes comprised 25.19% of the pan-genome, while the combined shell and cloud genes (which together make up the accessory genome) comprised 74.81% of the pan-genome. We identified 1716 genes, representing 22.88% of the species pan-genome, that are unique to a single strain.

The maximum likelihood phylogenetic tree based on the alignment of 89,143 SNPs of the core genes revealed many deep branching lineages consisting of 31 previously identified STs (Fig. 1A). The most common STs were ST5 (*n* = 15 genomes), ST8 (*n* = 7 genomes), ST30 (*n* = 8 genomes), ST133 (*n* = 7 genomes) and ST2187 (*n* = 16 genomes). Despite the unequal number of isolates from the four states (Fig. 1B), the phylogeny showed a lack of structure relative to the state from which the isolate originated.

Some STs were obtained from multiple animal host species (Fig. 1C), indicating a broad host range: ST5 in cat (*n* = 3), dog (*n* = 8), bird (*n* = 4); ST8 in cat (*n* = 4), dog (*n* = 3); ST30 in cat (*n* = 3), dog (*n* = 3), horse (*n* = 1), reptile (*n* = 1); ST133 in horse (*n* = 3), dog (*n* = 1), goat (*n* = 1), rabbit (*n* = 1), cow (*n* = 1); ST398 in horse (*n* = 3), bird (*n* = 1), cow (*n* = 1), goat (*n* = 1). STs of which all isolates were associated with only a single animal species included STs 151, 352 and 2187, which all originated from cows. However, this ST distribution among animal hosts may reflect the disproportionate number of isolates from cattle in our dataset and is not necessarily reflective of host-restricted niches.

### Distribution of acquired genes related to antibiotic resistance

**Figure 2:**
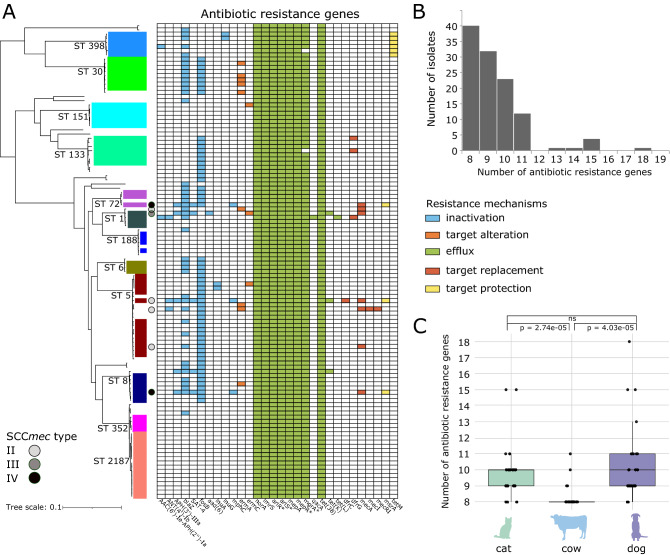
Antibiotic resistance profiles of animal-associated *S. aureus* in New England. (**A**) Distribution of acquired antibiotic resistance genes. The genes are color-coded according to resistance mechanisms. The type of SCC*mec* is also shown. Asterisks indicate genes associated with antibiotic resistance regulation. (**B**) Number of antibiotic resistance genes per genome. (**C**) Comparison of number of resistance genes per *S. aureus* genome in cats, cows and dogs. Significance was estimated using Welch’s t-test.

We determined the presence of acquired genes associated with resistance to different antibiotics. These genes represent a variety of resistance mechanisms (drug inactivation, target alteration, efflux, target replacement, target protection) based on definitions in the CARD database^17^. We identified a total of 30 resistance genes across the entire dataset (Fig. 2A and Supplementary Table S3).

In silico detection of the *mecA* gene from the genome sequences revealed seven MRSA isolates and 107 methicillin-susceptible *S. aureus* (MSSA) isolates. The *mecA* gene encodes an extra penicillin-binding protein (PBP2a) that has low affinity to virtually all beta-lactam antibiotics^18,19^. The *mecA* gene is carried by a mobile chromosomal cassette SCC*mec* and is classified into types based on the combination of the *ccr* and *mec* complexes they carry^19^. To date, there are 14 structurally distinct SCC*mec* types (I–XIV) that have been described^19–21^. SCC*mec* typing of the New England *S. aureus* population revealed the presence of types II (*n* = 4 genomes), III (*n* = 1 genomes) and IV (*n* = 2 genomes) (Fig. 2A and Supplementary Table S1). SCC*mec* type II was detected in ST 5, type III in ST1 and type IV in STs 8 and 72. We did not detect the gene *mecC*, which is a divergent form of *mecA* and also mediates beta-lactam resistance^22^. We found a slight discrepancy between the in silico detection of the *mecA* gene and in vitro phenotypic testing for methicillin resistance. There were two isolates (3659B and 4052C) whose genomes did not contain the *mecA* gene but were phenotypically tested as MRSA. These alternative mechanisms warrant further study.

Resistance-related genes associated with antibiotic efflux systems were ubiquitous in our dataset. We identified 11 such acquired genes, of which seven were found in all 114 genomes. These seven genes included *norA* (fluoroquinolone resistance)*, tet(38)* (tetracycline resistance), *arlRS* (fluoroquinolone resistance)*, lmrS* (multidrug resistance) and *mepAR* (multidrug and tigecycline resistance). The expression of *norA* is affected by the two-component regulatory system ArlRS^23^. *lmr*S confers resistance to aminoglycosides, macrolides, phenicols, diaminopyrimidine and oxazolidinone^24^. *mep*A is an efflux protein regulated by *mep*R and part of the *mep*RAB cluster, which makes up the multidrug and toxin extrusion (MATE) mechanism in *S. aureus*^25^. Also widely detected was *mgrA* (also known as *norR*), which is a regulator for *norA* and *tet(38)*^26^. The *mgrA* gene was detected in all genomes except two.

Other resistance genes were less frequently detected. The gene *fosB* (fosfomycin resistance) was detected in 60 genomes and was present in some genomes from STs 5, 6, 8, 30, 72 and 133. We detected the gene *blaZ* (beta-lactam resistance) in 43 genomes and was present in some genomes from STs 5, 6, 8, 30, 72 and 398. In all, every genome carried at least eight resistance-related genes, of which seven genomes have at least 13 resistance related genes (Fig. 2B). Among these less common genes, their distribution across disparate parts of the phylogeny indicates the potential for multiple independent HGT events across different genetic backgrounds, rather than the spread of resistance through clonal expansion.

We next compared the frequency of resistance-related genes among isolates from different animal species. To maintain consistency in our comparison, we focused only on those animals with the highest number of isolates (cats, cows, dogs). We detected a median of ten resistance-related genes (range = 8–15) in *S. aureus* from cats, ten resistance-related genes (range = 8–18) in *S. aureus* from dogs and eight resistance-related genes (range = 8–11) in *S. aureus* from cows (Fig. 2C). The *aad(6)* gene (aminoglycoside resistance) was found only in one isolate from a cat, whereas the resistance genes *dfrC* (diaminopyrimidine resistance), *lnuG* (lincosamide resistance), *mecI* and *mecR1* (beta-lactam resistance), and *tetM* (tetracycline resistance) appeared only in isolates from dogs. Several other resistance genes including *mecA, mphC* (macrolide resistance)*, msrA* (erythromycin and streptogramin resistance)*, tetK* (tetracycline resistance)*,* APH(3’)-IIIa (aminoglycoside resistance)*, ermA* (resistance to streptogramin, macrolide and lincosamide) and SAT-4 (nucleoside resistance) were present in *S. aureus* from dogs and cats, but not from cows. Three STs (STs 151, 352, 2187) which were isolated exclusively from cows exhibited a lack of accessory antimicrobial resistance genes, except for *ermC* found in a single isolate in those three STs. All other resistance genes found in these three STs were core genes found across all other genomes in the study. The total number of resistance genes varied between isolates from cats and cows, as well as between isolates from dogs and cows (p = 2.74e-05 and 4.03e-05, respectively; Welch’s t-test), but not between isolates from dogs and cats (p = 0.527; Welch’s t-test) (Fig. 2C).

### Distribution of staphylococcal virulence genes

**Figure 3:**
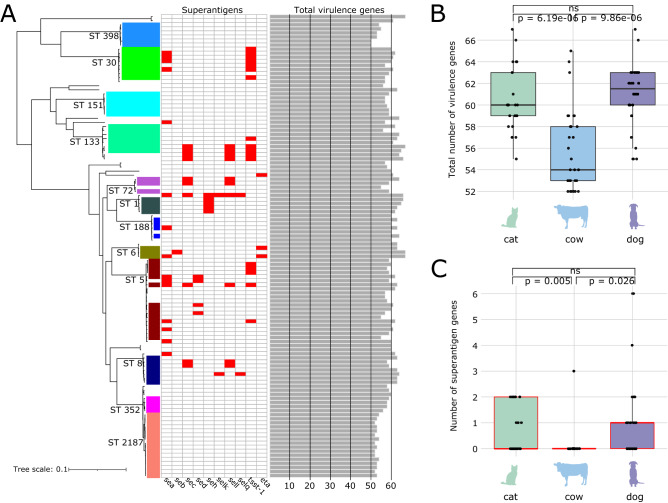
Virulence profiles of animal-associated *S. aureus* in New England. (**A**) Phylogenetic distribution of superantigen genes (shown in presence [red] or absence [white] matrix) and total number of virulence genes (shown in bar plots). (**B**) Number of virulence genes per *S. aureus* genome in cats, cows and dogs. (**C**) Number of superantigen genes per *S. aureus* genome in cats, cows and dogs. Significance was estimated using Welch’s t-test.

Animal-associated *S. aureus* carry numerous virulence-related genes. We detected 80 virulence genes in all 114 genomes and of which ten are known as superantigens (Fig. 3A and Supplementary Table S4). Superantigens constitute a family of secreted toxins that trigger excessive non-specific T-cell activation and proliferation, resulting in the overproduction of cytokines^27^. These potent toxins cause a variety of human diseases from transient food poisoning to lethal toxic shock^27^. To date, there are at least 24 known superantigens in *S. aureus*^28^. Of the 114 genomes in our dataset, 39 genomes harbor 1–6 distinct superantigens that were distributed in divergent parts of the phylogeny. Similar to the phylogenetic distribution of the antibiotic resistance genes described above, the distribution of the superantigens across diverse STs or lineages suggest multiple independent HGT events. The most common superantigen genes were *tsst-1* (*n* = 17 genomes in STs 5, 30 and 133), *sea* (*n* = 15 genomes in STs 5, 6, 30 and 188) and *sec* (*n* = 10 genomes in STs 5, 8, 72 and 133). STs 151, 352, and 2187 which were isolated exclusively from cows contained no superantigen genes, while ST 2187 exhibited fewer virulence genes compared to all other genomes. ST 398, collected from diverse animal hosts, also showed fewer virulence genes and was additionally void of superantigen genes. A notable virulence factor in *S. aureus* is the Panton-Valentine Leukocidin (PVL) pore-forming cytotoxin assembled by the genes *lukF-PV* and *lukS-PV*^29^. PVL toxin-producing strains causes leukocytolysis and tissue necrosis and are often associated with community acquired MRSA infections in humans^30^. In our dataset, we detected 96 and 3 genomes that carry *lukF*-*PV* and *lukS-PV*, respectively.

We also compared the number of virulence genes among isolates from the three most common animal hosts. Staphylococcal virulence genes were unevenly distributed among the three animal hosts. Isolates from cats harbored a median of 60 virulence genes (range = 55–67), 62 (range = 55–67) in dogs and 54 (range = 52–65) in cows (Fig. 3B). The total number of virulence genes varied between strains from cats and cows, as well as between isolates dogs and cows (*p* = 6.19e-06 and 9.86e-06 respectively; Welch’s t-test), but not between isolates from dogs and cats (*p* = 0.331; Welch’s t-test) (Fig. 3B). When superantigens were considered, the number of superantigen genes carried by a genome ranged from 0–2, 0–3 and 0–6 in isolates from cats, cows and dogs, respectively (Fig. 3C). There was also no significant difference in the number of superantigen genes between strains from dogs and cats (*p* = 0.366; Welch’s t-test). Three superantigen genes (*sec*, *sell*, *tsst-1*) were found in isolates from dogs, cats and cows. Six superantigen genes (*eta*, *sea*, *sed*, *seh*, *selk*, *selq*) were found only in dogs and cats, but not cows. Lastly, *seb* was found only in a single dog.

### Widespread gene sharing between animal-associated *S. aureus*

**Figure 4:**
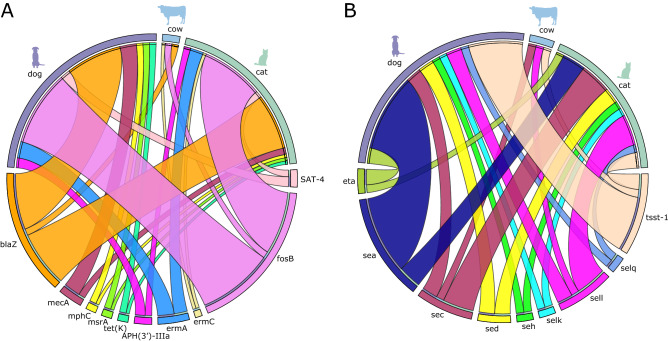
Distribution of antibiotic resistance (**A**) and superantigen (**B**) genes in *S. aureus* from cats, cows, and dogs. Only genes present in more than one isolate are shown and only the three most common animals were included. The outer ring of the upper half of circos plots represent the number of isolates sampled from dogs (purple), cows (light blue), and cats (light green). The outer ring of the bottom half of the circos plots represent the number of isolates that carry the shared antibiotic resistance genes (panel A) and superantigen genes (panel B). Connecting lines between the specific gene and the animal host are shown if the gene was detected in isolates from any of the three animal hosts.

Evidence for HGT in *S. aureus* and its contributions to adaptation in animal hosts has been demonstrated previously^16,31,32^. However, little is known about the spread of potentially transferrable resistance and superantigen genes among the animal hosts of *S. aureus*. We mapped the presence or absence of these genes shared between isolates from the three most common animal hosts (cats, cows, dogs). For the 15 resistance genes found in more than one isolate, the genes *blaZ* and *fosB* were present in isolates from all three animal hosts (Fig. 4A). We detected *blaZ* in 2, 17 and 17 isolates from cows, cats and dogs, respectively. We detected *fosB* in 2, 19 and 23 isolates from cows, cats and dogs, respectively. The genes which were detected in isolates from both cats and dogs, but not cows, were APH(3')-IIIa, *ermA*, SAT-4, *mecA, mphC, msrA*, and *tet(K).* The gene *ermC* was present in isolates from cats and cows, but not dogs. Lastly, there were no genes that were shared between dogs and cows, but not cats.

Staphylococcal superantigens genes are often located in mobile genetic elements, such as prophages, transposons, plasmids and pathogenicity islands^33,34^, thus facilitating their mobilization. We found that superantigen genes were widely distributed among isolates from different animal hosts (Fig. 4B). We detected *sec* in 1, 4, and 2 isolates from cows, cats and dogs, respectively. We detected *tsst*-1 in 1, 2, and 7 isolates from cows, cats and dogs, respectively. The genes *sea*, *sed*, *seh*, *selk*, *selg*, and *eta* were present in isolates from both dogs and cats, but not cows. No superantigens were shared between isolates from dogs and cows, but not cats, Lastly, no superantigen genes were shared between isolates from cats and cows, but not dogs. Overall, these results showed widespread gene sharing between *S. aureus* gene pools from different host species.

## Discussion

While humans are considered its primary reservoir, *S. aureus* can readily cross species barriers and infect new hosts^16^. Host-jumping events of bacterial pathogens are likely amplified by agricultural intensification, habitat encroachment and animal domestication^35,36^. In the case of *S. aureus*, the range of eukaryotic species that it can colonize as a commensal or opportunistic pathogen remains unclear. Regardless, the remarkable capacity of *S. aureus* to adapt to new or multiple hosts thus makes it a formidable bacterium that can threaten animal health, agriculture and the economy. Host-jumping events are often associated with acquisition of genetic elements from host-specific gene pools that confer traits required for survival and adaptation in the new host niche^16^. These traits include a variety of virulence factors such as superantigens that can be used to manipulate innate and adaptive immune responses^28^. For example, HGT in *S. aureus* mediated by mobile genetic elements occurs rapidly after a host-jumping event, potentially affecting the innate immune response of the new host^16^. During animal colonization, frequent HGT is facilitated by few genetic barriers to HGT in vivo (e.g., restriction–modification systems) and the successful replication and integration of different mobile elements^37,38^.

Here, we sought to elucidate the population genomic structure of 114 *S. aureus* isolates sampled from diseased animals in New England, USA from 2017–2019. We found that many multidrug resistant STs were detected in multiple wild and domestic animals. Notable were STs 5, 8 and 30, which are major *S. aureus* clones that are implicated in nosocomial and community-associated infections in humans^3,39^. ST133 isolates has been identified in healthy donkeys destined for food consumption in Tunisia^40^, caprine and ovine animals in Australia^41^, and the gut of healthy humans in Spain^42^. ST2187 has a long history of association with cows, and more specifically their milk^43,44^. Although some *S. aureus* lineages are specifically adapted to a narrow host range on a short evolutionary time scale^45^, as in the case of STs 151, 352 and 2187 in our study, this may be due to uneven and scarce sampling done in animals, particularly of wildlife species. Also notable is that 43 out of 114 genomes from six STs presented *blaZ*. For comparison, high penicillin susceptibility rate has been reported in MSSA from bloodstream infections in humans, primarily from CCs 5 and 398^46^. Penicillin may be considered a therapeutic option in the treatment of animal infections, but ST identity should be carefully considered as *blaZ* appears to be more phylogenetically widespread in animal-associated *S. aureus*. As for the two isolates whose genomes did not contain the *mecA* gene but were phenotypically tested as MRSA, a similar finding has been reported in four isolates from the Scottish MRSA Reference Laboratory and which has been posited to indicate the existence of alternative mechanisms of beta-lactam resistance^47^. Whole genome sequencing also revealed that those isolates phenotypically tested as MSSA often harbor numerous antibiotic resistance determinants. This result highlights the need to further investigate resistance characteristics beyond methicillin resistance in animal-associated *S. aureus*, which are often overlooked in many surveillance studies. In addition, such efforts will be instrumental in advancing the One Health concept, focused on the interconnectedness of animal, human and environmental well-being^10^.

Genes that encode for antibiotic resistance and superantigens were shared not only between divergent genetic backgrounds (or STs) of *S. aureus* but also between the animal hosts in which they reside. Hence, the ability of *S. aureus* to colonize multiple animal hosts means that mobilizable DNA may be disseminated more widely, creating a shared pool of resistance and virulence that is not limited by the animal hosts that harbor them. Our study also showed that cats, cows and dogs were frequent carriers of numerous *S. aureus* clones, each with distinct repertoire of resistance and superantigen genes. Hence, these animals are a major reservoir of clinically relevant genes and high-risk clones that can be transmitted to humans through frequent close contact. Overlapping ecological niches and/or physical proximity between animal hosts (e.g., pets in the same household, livestock animals in the same or nearby farms, interactions at the interface between wild and domestic animals) can certainly promote multi-host colonization and frequent gene sharing between *S. aureus* isolates. Domestic animals can therefore act as melting pots whereby genetic elements from various *S. aureus* lineages are combined in new genetic backgrounds. A variety of genetic assortments can promote the rapid emergence of high-risk clones with novel phenotypic characteristics. Large-scale genomic changes derived through HGT can generate “hopeful monsters” that may potentially cause public and animal health threats in ways that are hard to predict^48,49^. Because *S. aureus* can also acquire DNA from other *Staphylococcus* species with which it shares its niche with^31,50^, its gene pool may be further augmented with DNA that can confer additional adaptive or pre-adaptive features.

There are limitations in our study that need to be acknowledged. We recognize the sampling bias in our dataset that heavily favored isolates from cats, cows and dogs. Moreover, most isolates in our dataset were collected in New Hampshire where NHVDL is located, resulting in comparatively low sample sizes from the surrounding regions. Such bias did not allow us to carry out a more systematic analyses of the host distribution of STs and patterns of gene sharing between animal hosts that included other eukaryotes. The limited number of eukaryotic hosts means that those STs identified as host-restricted may in fact be found in multiple animal species. This also means that certain STs were overrepresented and rare ones were overlooked. Wildlife-associated *S. aureus* may likely harbor novel genetic variants or mobile genetic elements that pose an unknown level of risk to humans, companion animals and livestock. The structure and gene content of mobile genetic elements, such as pathogenicity islands and phages, and how they shape patterns of gene sharing in animal-associated *S. aureus* should also be investigated. Future work should therefore include a broader surveillance of *S. aureus* in other less commonly studied domestic animals and wildlife species, especially those species that often interface with livestock and/or exist at the junction of urban and natural landscapes. It is likely that the known range of species that *S. aureus* can colonize will expand as studies continue to examine *S. aureus* in wild animals. Our dataset also included only isolates from disease cases; hence, we do not have data to describe the extent of *S. aureus* carriage in animals and its contributions to the overall population genetic structure. Comparison of *S. aureus* in carriage and infections is critical to understanding the genetic basis of pathogenicity and hence, reduce the threats to animal health. Lastly, our study encompassed only three years of bacterial sampling, which was not sufficient to elucidate the long-term evolution of highly virulent and/or multidrug resistant lineages. Future investigations therefore necessitate close monitoring of high-risk clones and the underlying reasons that contribute to their persistence or expansion in the population.

In summary, our findings highlight the role of animals in disseminating resistance and virulence determinants of *S. aureus*. The remarkable ability of *S. aureus* as a versatile, multi-host pathogen lies partly on its ability to acquire and disseminate genetic material between lineages and between animal hosts within a short period of time. This study reveals widespread gene sharing between bacterial strains colonizing different animal hosts and highlights the need for routine surveillance to capture the dynamic genetic context of *S. aureus*.

## Methods

### Sample collection in New England

The New England *S. aureus* collection consisted of 133 isolates that were retrospectively sampled from September 2017 through March 2020. Isolates were obtained as culture swabs from routine clinical specimen submissions to the New Hampshire Veterinary Diagnostic Laboratory (NHVDL), New Hampshire, USA. The clinical specimens were received from multiple veterinary practices from the states of Connecticut, New Hampshire, Maine, Massachusetts and Vermont. These states are located in the northeastern part of the country. All isolates were from animals with confirmed clinical infections. No live vertebrates were used in this study; hence, the NHVDL was exempt from the IACUC approval process. Pure isolates were cultured in commercially prepared tryptic soy agar with 10% sheep red blood cells and brain heart infusion broth. Initial species identification was carried out using matrix-assisted laser desorption/ionization time-of-flight mass spectrometry (MALDI-TOF MS) using the Bruker Biotyper instrument and following manufacturer’s protocols. Species assignments were made by comparing mass spectra of our samples to two libraries of reference spectra RUO library 6903(V6) and 7311(V7) available in the Bruker MBT Compass (Bruker Daltonics, Bremen, Germany). The most common sites of isolation included skin, ears, nasal passages, milk and wounds. Associated metadata information for each isolate are included in Supplementary Table S1. All isolates were stored in DMSO solution in − 80 °C.

### Methicillin susceptibility screening

In vitro screening for cefoxitin and oxacillin resistance was carried out using the Kirby Bauer disc diffusion technique. We followed the breakpoint guidelines of the most current Clinical and Laboratory Standards Institute using cefoxitin, which is used as the official predictor of methicillin resistance for *S. aureus*^51^. For isolates identified as methicillin resistant, we determined the presence of the penicillin-binding protein PBP2 using a commercial latex agglutination test (MASTALEX MRSA Latex Kit, MAST, UK) following manufacturer's guidelines. We confirmed the presence of the *mecA* gene by screening the genome sequence of each isolate (described below).

### DNA extraction and whole genome sequencing

DNA extraction was carried out using the Zymo Research Quick-DNA Fungal/Bacterial Miniprep Kit (Irvine, California) following the manufacturer's protocol. We quantified DNA concentration using a Qubit fluorometer (Invitrogen, Grand Island, NY). We prepared DNA libraries using the Nextera XT protocol with 1 ng of genomic DNA per isolate. Samples were sequenced using the Illumina HiSeq platform (San Diego, California) to produce 250 bp paired end reads. Sequencing was carried out at the Hubbard Center for Genome Studies at the University of New Hampshire.

### Genome assembly and annotation

We assembled all genomes using Shovill v1.1.0 (https://github.com/tseemann/shovill). Shovill is a series of methods that includes subsampling read depth down to 150X, trimming adapters, correcting sequencing errors and assembling using SPAdes v3.13.0^52^. We used QUAST v5.0.2^53^ and CheckM v1.1.3^54^ to assess the quality of our sequences and exclude genomes with < 90% completeness and > 5% contamination. We also excluded assemblies with > 200 contigs and an N50 < 40,000 bp. Only 19 out of 133 genomes did not pass these quality thresholds and were therefore excluded from all downstream analyses. The remaining 114 genomes were annotated using Prokka v1.14.6^55^. Genomes were compared to the *S. aureus* reference genome (NCBI Reference Sequence: Accession No. NC_007795.1) with the program FastANI v1.32 to confirm species identification using the > 95% average nucleotide identity (ANI) threshold^56^.

### Pan-genome analysis and phylogenetic reconstruction

We used Roary v3.13.0^57^ to characterize core genes and accessory genes that make up the pan-genome^57^. To balance the tradeoff between inferring robust phylogenetic relationships versus accounting for assembly errors, we included core genes if they were present in ≥ 99% of the genomes. Nucleotide sequences of each orthologous gene family were aligned using MAFFT v7.475^58^. Aligned core genes were concatenated to generate a core genome alignment. Phylogenetically informative single nucleotide polymorphisms (SNPs) in the core genome alignment were extracted using SNP-sites^59^. We used the core SNP alignment to construct a maximum likelihood phylogenetic tree using RAxML v8.2.12^60^ employing a general time-reversible nucleotide substitution model^61^ and four gamma categories for rate heterogeneity. Phylogenetic trees were visualized using the online platform Interactive Tree of Life (IToL)^62^.

### In silico sequence typing, detection of resistance genes, virulence genes and SCC*mec*

Using the contig files, we determined the multilocus sequence types (ST) for all genomes used in this study using the program mlst v2.19.0 (https://github.com/tseemann/mlst). STs pertain to allelic profiles that characterize nucleotide differences in partial sequences of seven housekeeping genes^63^. In *S. aureus*, these seven genes consist of *arcC, aroE, glpF, gmk, pta, tpi* and *yqiL*^64^. Allelic profiles of the genomes used in this study were compared to those in the *S. aureus* MLST database (https://pubmlst.org/)^65^. We screened for the presence of horizontally acquired antibiotic resistance genes and virulence factors using ABRicate v1.0.1 (https://github.com/tseemann/abricate) utilizing the Comprehensive Antibiotic Resistance Database (CARD)^17^ and the Virulence Factor Database (VFDB)^66^. Finally, we used SCCmecFinder^67^ implemented in SCCion v0.1 (https://github.com/esteinig/sccion) to determine the presence and type of the mobile genetic element SCC*mec*. We used the minimum thresholds of > 60% for sequence coverage and > 90% sequence identity to identify the SCC*mec*. Visualization of the distribution of resistance and virulence genes was carried out using Circos^68^.

We used the default parameters for each program unless indicated otherwise.

### Statistical tests

We used Welch’s t-test to compare the number of resistance and virulence genes of isolates from different animal hosts. Results were considered significant when *p* < 0.05.

## Supplementary Information

Supplementary Information.

## Data Availability

Raw reads of the 114 *S. aureus* genomes have been deposited in the Sequence Read Archive (SRA) in the National Center for Biotechnology Information (NCBI) under BioProject accession No. PRJNA741582. Sample accession numbers and associated metadata are listed in Supplementary Table S1.
